# 

**Published:** 1888-07

**Authors:** 


					﻿Volume LVIL]
JULY, 1888
I Number I.
ssgS≥
ifol al BJ LCil M
I äfr/r £1 11CI ■! ri
Rand, McNally & Company, Printers.
1888.
Enteredat trie Pott Office at Chicago, for trantmiteion through the mail* at leoend-olatt matter.
				

